# An alternative fully human anti-BCMA CAR-T shows response for relapsed or refractory multiple myeloma with anti-BCMA CAR-T exposures previously

**DOI:** 10.1038/s41417-023-00712-0

**Published:** 2023-12-15

**Authors:** Qingming Wang, Runhong Wei, Shufang Guo, Chao Min, Xiong Zhong, Hui Huang, Zhi Cheng

**Affiliations:** 1 Department of Hematology, The Second Affiliated Hospital of Nanchang University, Nanchang, Jiangxi China; 2 Department of Hematology, Henan Province Hospital of Traditional Chinese Medicine (The Second Affiliated Hospital of Henan University of Traditional Chinese Medicine), Institute of Hematology, Henan University of Traditional Chinese Medicine, Zhengzhou, China; 3 Nanchang University, Nanchang, Jiangxi China; 4 HRAIN Biotechnology Co., Ltd., Shanghai, China

**Keywords:** Tumour immunology, Cancer immunotherapy, Cancer immunotherapy

## Abstract

Chimeric antigen receptor T (CAR-T) cells therapy has made remarkable progress in relapsed/refractory multiple myeloma (R/R MM) treatment. Unfortunately, patients still eventually experience disease progression or relapse even after receiving anti-BCMA CAR-T therapy. At present, there are limited data on available treatment options for patients who have progressed on anti-BCMA CAR-T therapy. In this study, we evaluated the safety and efficacy of fully human anti-BCMA CAR-T (HRC0202) in seven R/R MM patients who were previously exposed to anti-BCMA CAR-T therapy. Three patients received 6.0 × 10^6^ CAR^+^T cells/kg, one patient received 10.0 × 10^6^ CAR^+^T cells/kg and three patients received 15.0 × 10^6^ CAR^+^T cells/kg. Cytokine release syndrome (CRS) of grades 1–2 occurred in three patients (42.9%) and grade ≥3 in two patients (28.6%). Immune effector cell-associated neurotoxic syndrome (ICANS) was not observed in any of the patients. The best overall response rate (ORR) was 71.4% (5/7), with a stringent complete response/complete response (sCR/CR) achieved in three patients. The median progression-free survival (PFS) was 269 days, and median overall survival (OS) for all patients was not reached. The median peak concentration (C_max_) of HRC0202 was 30117.70 (range, 6084.35–147415.10) copies/μg DNA. This study indicated that fully human anti-BCMA CAR-T (HRC0202) is a promising treatment for R/R MM patients who relapsed or refractory from prior anti-BCMA CAR-T infusion.

## Introduction

Multiple myeloma (MM) is a plasma cell neoplasm characterized by clonal plasma cells that produce a monoclonal immunoglobulin. It accounts for 1% of all cancers and ~10% of all hematologic malignancies [1]. In recent years, with the clinical application of proteasome inhibitors (PIs), immunomodulatory drugs (IMiDs), monoclonal antibodies (mAbs), histone deacetylase inhibitors and nuclear export inhibitors, the survival of MM patients was significantly improved, but MM remains an incurable disease [2–6]. The emergence of CAR-T cell therapy has brought new hope for MM treatment [7], B-cell maturation antigen (BCMA) is a protein that is commonly found on the surface of multiple myeloma cell. To date, the US Food and Drug Administration (FDA) has approved two anti-BCMA CAR-T therapies, including Abecma (Idecabtagene vicleucel, ide-cel) and Carvyti (Ciltacabtagene autoleucel, cilta-cel), and China National Medical Products Administration (NMPA) has approved one anti-BCMA CAR-T therapy Fucaso (Equecabtagene autoleucel, CT103A) for the treatment of R/R MM. At a median follow-up of 13.3 months, the ORR of ide-cel was 73% (94/128), with 33% patients (42/128) achieving a strict complete response/complete response (sCR/CR), the median progression-free survival (PFS) and overall survival (OS) were 8.8 months and 19.4 months, respectively [8]. At a median follow-up of 12.4 months, the ORR of cilta-cel was 97% (94/97), with 67% patients (65/97) achieving strict complete response, the 27-month PFS and OS rates were 54.9% and 70.4%, respectively [9]. At a median follow-up of 13.8 months, the ORR of CT103A was 96% (97/101), with 74.3% patients (75/101) achieving sCR/CR, the median duration of response (DOR) and PFS have still not reached [10].

More than 50% patients are likely to relapse within 12 months [11, 12], and treatment options are limited and there is no established standard of care for these patients. In a clinical study of sixty-eight patients who experienced disease progression after receiving ide-cel as part of the KarMMa study, the median duration of the subsequent treatment regimen was a mere 44 days [13]. Deep responses were reported in four prior murine anti-BCMA CAR-T exposure patients who received a fully human BCMA-targeting CAR-T treatment [14]. In a recent study, seventy-nine patients who experienced relapse after receiving BCMA-directed CAR-T therapy were analyzed, and the median PFS for the first attempted salvage therapy was 3.5 months. Moreover, out of these patients, 35 received a subsequent T-cell-engaging therapy, including CAR-T or bispecific antibody treatment, the objective response rate was 91.4%, and the median OS was not reached. Those initial evidence suggest that alternative anti-BCMA treatment can be effective while there is currently no established best strategy for patients who were previously exposed to anti-BCMA CAR-T therapy.

A thorough assessment of patients’ outcomes following anti-BCMA CAR-T is critical in establishing a benchmark for clinical trials and determining the optimal treatment strategy for those patients. In this study, we investigated the safety and efficacy of the fully human anti-BCMA CAR-T (HRC0202) in seven R/R MM patients who were previously exposed to anti-BCMA CAR-T therapy.

## Materials and methods

### Study design and patients

This study is a phase 1, open-label, 3 + 3 dose-escalation clinical trial, the objective is to study the safety and efficacy of anti-BCMA CAR-T cells in treating patients with MM. The study is performed in the second affiliated hospital of Nanchang University and the second affiliated hospital of Henan University of Traditional Chinese Medicine, and has registered in ClinicalTrials.gov (NCT04003168). In this paper, R/R MM Patients relapsed or refractory from prior anti-BCMA CAR-T infusion were included and analyzed.

The major eligibility criteria included diagnosis of MM by IMWG 2014; patients previously received at least three-line treatment regimens for multiple myeloma, including protease inhibitors and immunomodulators, and have disease progression within 60 days of the latest treatment; clinical performance status of ECOG score 0–1; adequate organs function to tolerate this therapy. The major exclusion criteria included patients have a history of central nervous system (CNS) disease; active hepatitis B or C, HIV and syphilis infections; the terminal organ damaged due to autoimmune diseases, or the systemic use of immunosuppressive or other systemic disease control drugs was required; active systemic infections or uncontrolled infection (for detailed inclusion and exclusion criteria, please refer to the ClinicalTrials.gov). In addition, patients received murine CAR-T treatment previously, were enrolled based on investigator’s evaluation of the risks and benefits of patients receiving the experimental drug.

This study was conducted in accordance with the Declaration of Helsinki and the International Conference on Harmonization guidelines for Good Clinical Practice, and the study protocol was approved by institutional review board for Henan Province Hospital of Traditional Chinese Medicine (No. 2018-KI-024-01).

### CAR-T production and administration

HRC0202 was generated by HRAIN Biotechnology (Shanghai, China). Patients would receive bridging chemotherapy during HRC0202 preparation by investigator’s evaluation. T cells were isolated from peripheral blood mononuclear cells (PBMCs) and transduced with a retrovirus vector encoding a second-generation CAR incorporating an extracellular single-chain variable fragment (scFv) from a fully human anti-BCMA antibody (HK10), a transmembrane region, and an intracellular fragment comprising the CD137 (4-1BB) costimulatory motif and the CD3-zeta (CD3ζ) signaling domain. CAR-T cells were stimulated with anti-CD3 and anti-CD28 antibodies and expanded as described previously [15]. After prepared and qualified quality control of CAR-T, patients underwent 3 days of lymphodepletion (cyclophosphamide 300 mg/m^2^/day and fludarabine 30 mg/m^2^/day), and the lymphodepletion dose could be given in the half if patients had grade ≥3 hematologic toxicities.

HRC0202 was infused intravenously on d0 at the dose of 6.0 × 10^6^ CAR^+^T cells/kg, 10.0 × 10^6^ CAR^+^T cells/kg or 15.0 × 10^6^ CAR^+^T cells/kg. Patients would receive 25 mg promethazine or 20 mg diphenhydramine as pre-medication to prevent infusion reaction ~30 min prior to the infusion.

### Detection of CAR-T cell and cytokines

The expression of BCMA CAR DNA in the peripheral blood was detected by quantitative polymerase chain reaction (qPCR) method on day −1, 1, 3, 5, 7, 10, 14, 21, 28, and subsequent follow-up point. Meanwhile, cytometric bead array (CBA) method was used to detect the cytokine levels of Granzyme B (560304, BD Biosciences), Interferon-γ (558269, BD Biosciences), Interleukin-6 (558276, BD Biosciences), Tumor necrosis factor (560112, BD Biosciences) in the peripheral blood, and enzyme-linked immunosorbent assay (ELISA) method was used to detect the cytokine levels of C-reactive protein (SCRP00B, R&D Systems), Interleukin-15 (S1500, R&D Systems) in the peripheral blood.

### Statistical analysis

The study reported descriptive statistics for continuous variables as mean ± standard deviation (SD) or median with range, and for categorical variables as counts and percentages. Categorical variables were evaluated using Chi-square tests, while continuous variables were compared using t-tests or Wilcoxon rank sum tests. The Kaplan-Meier method was used to estimate median PFS and OS. The difference was considered statistically significant if p <0.05.

## Results

### Patient characteristics

There were seven R/R MM patients, including four patients relapsed from prior anti-BCMA CAR-T infusion and three patients refractory from prior anti-BCMA CAR-T infusion. All patients had successfully prepared CAR-T cells and received HRC0202 infusion. Three patients received 6.0 × 10^6^ CAR^+^T cells/kg, one received 10.0 × 10^6^ CAR^+^T cells/kg and three patients received 15.0 × 10^6^ CAR^+^T cells/kg.

The median age of seven patients was 54 (range, 50–72) years. The type of monoclonal globulin was 4 IgG, 2 IgA, and 1 IgD. There were two patients with extramedullary disease (patient 5 had breast masses and patient 7 had dorsal masses). The median prior lines of therapy was 7 (range, 4–11). One patient (patient 1) previously received autologous hematopoietic stem cell transplantation (ASCT). Four patients received HRC0501 (murine-derived anti-BCMA/CD19 bispecific CAR-T), one patient (patient 3) received HDS269B (murine-derived anti-BCMA CAR-T), one patient (patient 1, previously reported [16]) received HDS269B and HRC0202 in previous therapy, one patient (patient 4) received HDS269B and HRC0501. The median proportion of myeloma cells in BM was 0.64% (range, 0.12–77.40%), and the median proportion of BCMA expression in myeloma cells was 2.65% (range, 0.00–89.01%). The median serum monoclonal protein level before HRC0202 infusion was 2.77 (range, 1.60–16.50) g/L (See Table 1).

**Table 1:** Baseline characteristics in 7 relapsed/refractory multiple myeloma patients.

Patient No.	Age (years)	Gender	Monoclonal Protein	Serum Monoclonal protein level (g/L)	Extramedullary disease	Previous lines	Previous ASCT	Previous CAR-T infusion	Previous CAR-T dose (×10^6^/kg)	Bone marrow plasma cells (%)	BCMA in plasma cells (%)	HRC0202 dose (×10^6^/kg)
1	51	Male	IgD-λ	2.55	No	7	Yes	HDS269B, HRC0202	HRC0202: 12	77.40	89.01	10
2	57	Male	IgG-κ	1.60	No	4	No	HRC0501	6	0.12	0.18	15
3	72	Female	IgA-κ	2.13	No	6	No	HDS269B	9	5.23	48.64	15
4	50	Male	IgG-κ	6.09	No	8	No	HDS269B, HRC0501	HRC0501: 6	0.67	0.00	15
5	57	Female	IgG-λ	2.77	Yes	4	No	HRC0501	6	0.19	2.00	6
6	54	Female	IgA-λ	16.50	No	11	No	HRC0501	6	0.64	17.07	6
7	51	Female	IgG-κ	4.95	Yes	7	No	HRC0501	3	0.13	2.65	6

The table shows study baseline characteristics, including demography, disease, previous treatment, previous CAR-T and HRC0202 infusion. ASCT, autologous hematopoietic stem cell transplantation; HDS269B: murine-derived anti-BCMA CAR-T; HRC0202: fully human anti-BCMA CAR-T; HRC0501: murine-derived anti-BCMA/CD19 bispecific CAR-T.

### Safety

All seven patients experienced treatment-emergent adverse events (TEAEs) after HRC0202 infusion. The most common TEAEs (incidence greater than or equal to 50.0%) included pyrexia (85.7%), leukopenia (71.4%), asthenia (57.1%), lymphopenia (57.1%), neutropenia (57.1%) and anaemia (57.1%), and CRS (71.4%). The most common Grade 3 or 4 TEAEs (incidence greater than or equal to 20.0%) included lymphopenia (57.1%), neutropenia (57.1%), leukopenia (42.9%), CRS (28.6%) (see Table 2).

**Table 2:** Hematologic and nonhematologic treatment-emergent adverse events (TEAEs).

TEAEs	Total, *N* = 7	
	Any grade	Grade 3–4
Hematologic TEAEs^a^		
Total patients with hematologic TEAEs^a^		
Leukopenia	5 (71.4)	3 (42.9)
Lymphopenia	4 (57.1)	4 (57.1)
Neutropenia	4 (57.1)	4 (57.1)
Anaemia	4 (57.1)	1 (14.3)
Thrombocytopenia	2 (28.6)	1 (14.3)
Nonhematologic TEAEs^a^		
Cardiac disorders		
Tachycardia	2 (28.6)	0
Gastrointestinal disorders		
Nausea	2 (28.6)	0
General disorders and administration site conditions		
Pyrexia	6 (85.7)	0
Asthenia	4 (57.1)	0
Fatigue	3 (42.9)	0
Pain	3 (42.9)	0
Investigations		
Blood lactate dehydrogenase increased	2 (28.6)	1 (14.3)
Blood alkaline phosphatase increased	2 (28.6)	0
Gamma-glutamyltransferase increased	2 (28.6)	0
Serum ferritin increased	2 (28.6)	0
Procalcitonin increased	2 (28.6)	0
C-reactive protein increased	2 (28.6)	0
Interleukin level increased	2 (28.6)	0
Respiratory, thoracic, and mediastinal disorders		
Chest discomfort		
Vascular disorders	2 (28.6)	0
Hypotension	2 (28.6)	0
Hypertension	2 (28.6)	
CRS and neurotoxicity		
CRS	5 (71.4)	2 (28.6)
Neurotoxicity	2 (28.6)	0

The table shows TEAEs by System Organ Class and Preferred Term. The most common TEAEs (incidence ≥ 50.0%) included pyrexia (85.7%), leukopenia (71.4%), asthenia (57.1%), lymphopenia (57.1%), neutropenia (57.1%) and anaemia (57.1%), and CRS (71.4%).

^a^Reported in ≥20% of patients.

CRS of any grade occurred in five patients (71.4%), and of grades 1–2 in three patients (42.9%), of grade ≥3 in two patients (28.6%). All CRS were resolved after symptomatic treatment, one patient was administered tocilizumab and dexamethasone, two patients were administered only tocilizumab, one patient was administered only dexamethasone and one patient didn’t receive tocilizumab or dexamethasone (Table 3). There was no ICANS observed in this study. Two patients experienced neurotoxicities, which were hypoaesthesia and dizziness, and they were reversible.

**Table 3:** Management of patients with CRS.

	CRS grading	Glucocorticoid	Tocilizumab
Patient 1	3	Yes	No
Patient 3	1	No	No
Patient 5	2	No	Yes
Patient 6	2	No	Yes
Patient 7	3	Yes	Yes

The table shows all CRS occurred after HRC0202 infusion and the use of tocilizumab and glucocorticoid.

### Efficacy

**Fig. 1:**
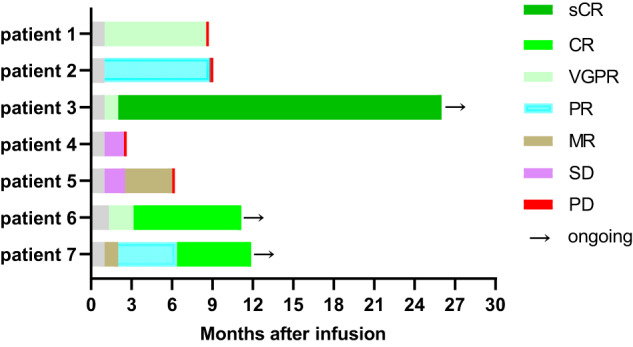
Clinical overall response after HRC0202 infusion. The bar chart shows the clinical overall response and follow-up of patients after HRC0202 infusion. Each bar represents an individual patient and corresponding number. The different colors represent different clinical response. sCR stringent complete response, CR complete response, VGPR very good partial response, PR partial response, MR minimal response, SD stable disease, PD progressive disease.

**Fig. 2:**
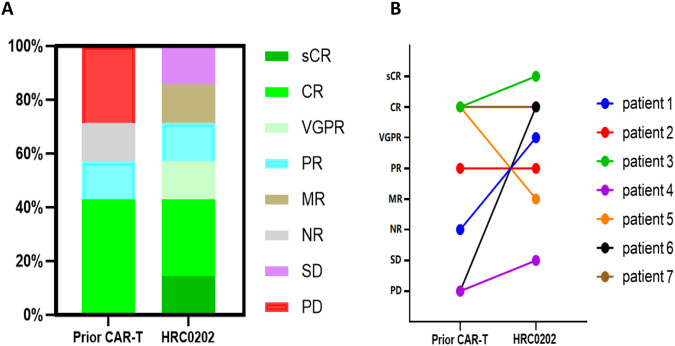
Best response to the prior CAR-T and HRC0202. (**A**) The chart shows different clinical response and their proportion to prior CAR-T and HRC0202. (**B**) The chart shows different patient and their best response to prior CAR-T and HRC0202. sCR stringent complete response, CR complete response, VGPR very good partial response, PR partial response, MR minimal response, NR no response, SD stable disease, PD progressive disease.

At a median follow-up time of 467 (range, 226–904) days, the best objective response rate (ORR) was 71.4% (5/7) with three patients (3/7, 42.9%) achieving sCR/CR, and two patients (2/7, 28.6%) achieving very good partial response (VGPR)/PR. Three patients had an ongoing response for >12 months, and one patient had an ongoing response for >25 months. After achieved the best response, five patients (5/7, 71.4%) showed MRD negative at 10^−4^ nucleated cells by flow cytometry. All of whom achieved CR, three patients (3/3, 100.0%) maintained CR for more than 8 months, and one patient (1/3, 33.3%) for more than 25 months (see Fig. 1). The median PFS was 269.0 (95% CI, 240.8–297.2) days, and median OS was not reached. Compared with the prior CAR-T infusion, HRC0202 infusion could still achieve considerable efficacy, four patients (57.1%) had a better response, two patients had the same response and one patient had a worse response (see Fig. 2). In addition, two patients with extramedullary disease can benefit from HRC0202 infusion, the best ORR for patient 5 was MR and patient 7 was CR in the follow-up.

### Pharmacokinetics and pharmacodynamics

**Fig. 3:**
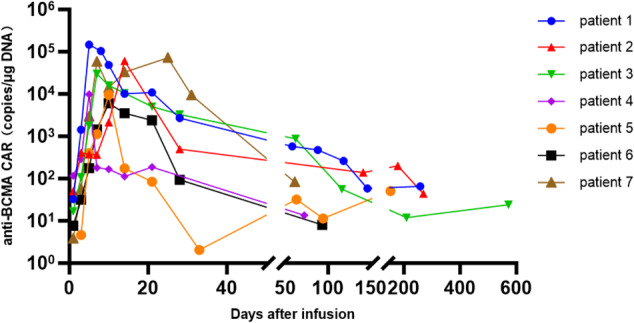
Pharmacokinetics of HRC0202. QPCR method was used to detect cell proliferation of HRC0202.

**Fig. 4:**
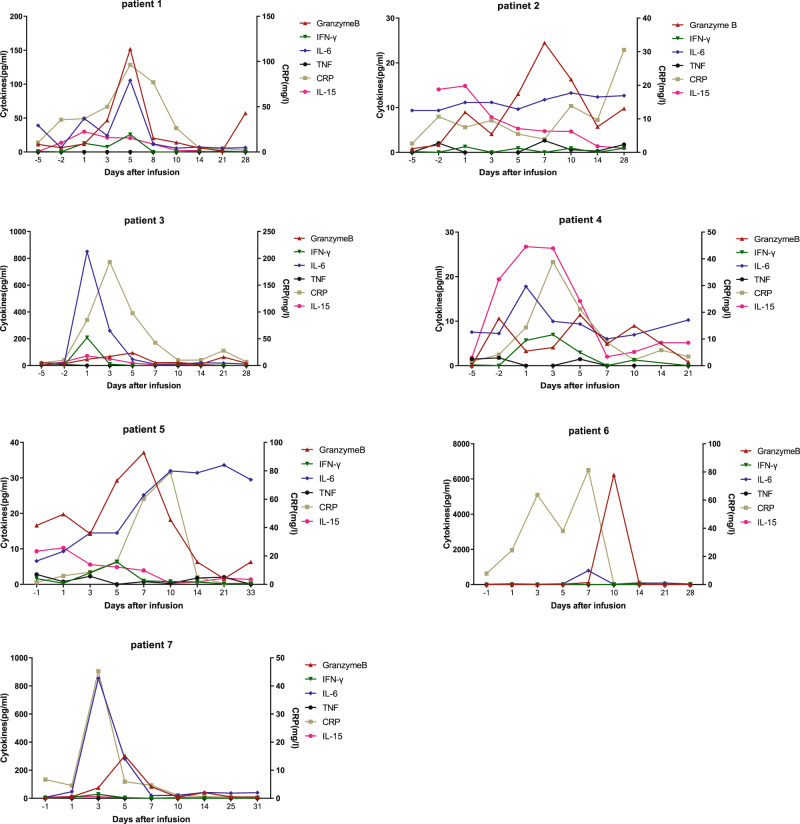
Cytokine levels in each patient. CBA and ELISA method were used to detect the cytokine levels before and after HRC0202 infusion. IFN-γ interferon-γ, IL-6 Interleukin-6, TNF Tumor necrosis factor, CRP C-reactive protein, IL-15 Interleukin-15.

The pharmacokinetics (PK) and pharmacodynamics (PD) of HRC0202 in the blood were monitored at serial time-points after CAR-T infusion in all seven patients (Figs 3 and 4). The median time to peak concentration (T_max_) was 10 (range, 5–25) days. The median C_max_ of BCMA CAR-T expansion was 30117.70 (range, 6084.35–147415.10) copies/μg DNA. The median AUC_0-28_ was 239466.00 (range, 23412.00-1084834.00) days×copies/μg DNA. The median values of HRC0202 infusion associated cytokines were 78.95 (range, 30.56–193.13) mg/L for CRP, 14.86 (6.10–50.04) pg/mL for IL-15, 94.60 (range, 11.42–6241.42) pg/mL for Granzyme B, 8.66 (range, 1.28–208.68) pg/mL for IFN-γ, 105.55 (range, 13.27–854.07) pg/mL for IL-6, 2.14 (range, 0.41–7.02) pg/mL for TNF-α.

## Discussion

Although R/R MM patients have achieved remarkable efficacy after received CAR-T treatment, there are more than 50% patients relapsed within 12 months, and no standard treatment has been established. Then patients are advised to receive clinical trials, previously sensitive drugs, unused new drugs, new combination treatment regimens or a second CAR-T infusion [17]. Some studies used murine CAR-T, human CAR-T and humanized CAR-T for repeat CAR-T infusion in R/R MM, and reported to be safe and effective [13, 14, 18, 19]. Due to the strict inclusion and exclusion criteria of CAR-T trials, patients who previously treated with CAR-T were outside of CAR-T clinical trials [20, 21], second infusion of anti-BCMA CAR-T cells have been considered as a possible approach to improve outcome, a potential concern is that the immunogenicity of CAR-T may limit efficacy if the same CAR-T as second infusion. In ide-cel (murine-derived anti-BCMA CAR-T) registered clinical trial, twenty-eight patients were retreated with ide-cel with a higher dose after progression, only 21% (6/28) had a second response, all six patients were antidrug antibody–negative [8]. Gazeau N et al. [18] reported a κ light chain MM patient didn’t response for secondly ide-cel infusion after relapse, the presence of anti-CAR antibodies at the time of relapse after the first infusion was contributed to an ∼26-fold lower expansion in the second infusion relative to the first infusion. Another concern is that reinfusion with the same CAR-T product had poor CAR-T expansion and efficacy as observation in B-cell acute lymphoblastic leukemia [22]. So, a different anti-BCMA CAR-T with an alternative scFv structure may be an optimism.

**Fig. 5:**
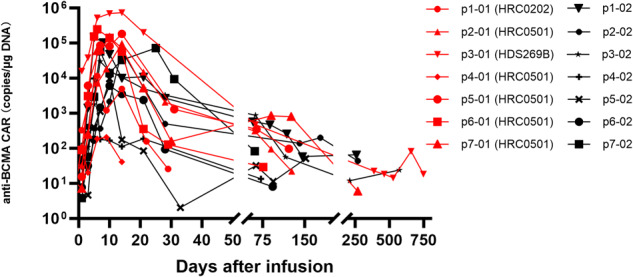
Comparison of pharmacokinetics between prior CAR-T and HRC0202. The chart shows cell proliferation of prior CAR-T and HRC0202. Median C_max_ of prior CAR-T was 142970.50 (range, 206.60–733066.20) copies/μg DNA compared with median C_max_ of HRC0202 was 30117.70 (range, 6084.35–147415.10) copies/μg DNA (p = 0.136).

In this study, a fully human anti-BCMA CAR-T (HRC0202) was infused to seven R/R MM patients who were relapsed or refractory from prior CAR-T infusion. Four patients received a higher CAR-T dose than prior CAR-T infusion, while two patients (patient 5 and patient 6) received the same CAR-T dose as prior CAR-T infusion and one patient (patient 1) received a lower CAR-T dose than prior CAR-T infusion. CRS occurred in five patients, and CRS didn’t appear to correlate with the dose as grade 3 CRS occurred in dose of 10.0 × 10^6^ CAR^+^T cells/kg (patient 1) and 6.0 × 10^6^ CAR^+^T cells/kg (patient 7), grade 2 CRS occurred in dose of 6.0 × 10^6^ CAR^+^T cells/kg (patient 5 and patient 6), grade 1 CRS occurred in dose of 15.0 × 10^6^ CAR^+^T cells/kg (patient 3). All CRS resolved with supportive treatment, tocilizumab and/or glucocorticoid, and ICANS was not observed in the trial. Infection of grades 1–2 occurred in two patients and no grade ≥3 infection. The best ORR was 71.4% (5/7), with a sCR/CR achieved in three patients (42.9%), and the median PFS was 269 days, and median OS was not reached. The median T_max_ was 10 days, the median C_max_ was 30117.70 copies/μg DNA, and the median AUC_0-28_ was 239466.00 days×copies/μg DNA. Further, we compared the pharmacokinetics of HRC0202 and prior CAR-T infusion and found that HRC0202 amplifications were lower than the prior CAR-T infusion (see Fig. 5), but there was no significant difference in C_max_ (*p* = 0.136) or AUC_0-28_ (*p* = 0.175). A robust immune response was reported to reduce CAR-T cells below detectable levels within 18–34 days after infusion [23], and the products of our study were different, so more study is need to explore the reasons. In addition, there were two patients (28.6%) with extramedullary disease (breast masses and dorsal masses, respectively), and after HRC0202 infusion, 1 had MR and 1 had CR in the follow-up efficacy evaluation. Therefore, extramedullary patients could benefit from HRC0202 infusion.

Clinical trials had supported the use of anti-BCMA CAR-T in R/R MM without restriction based on tumor BCMA expression [8, 9]. Fifteen of sixteen patients (94%) who relapsed after ide-cel infusion retained BCMA-expressing tumor cells in bone marrow, which indicates that those patients may benefit from anti-BCMA therapy. In this study, the median proportion of BCMA expression in myeloma cells was 2.65% (range, 0.00–89.01%), the efficacy of the BCMA expression negative patient (patient 4) was SD at 28-day and one month later was PD. However, biallelic genomic loss of BCMA on chromosome 16p was a resistance mechanism to anti-BCMA CAR-T therapy, which leads to lack of CAR-T cell proliferation following the second infusion [24]. These suggest the need for careful detection of BCMA gene alterations for reinfusion of CAR-T cell therapy if BCMA expression is negative.

Other approach for R/R MM patients include another targeted CAR-T and bispecific antibodies etc. In MagnetisMM-1 trial, 3 of 4 pts (75%) with prior BCMA-directed therapy achieved response (1 sCR, 2 VGPR) for Elranatamab, a humanized bispecific monoclonal antibody (BsAb) that targets BCMA [25]. In addition, treatment with teclistamab (a BCMA×CD3 BsAb) showed an ORR of 38% in anti-BCMA antibody drug conjugate-exposed patients and 45% in anti-BCMA CAR-T exposed patients [26]. Talquetamab (a GPRC5D×CD3 BsAb) had an ORR of 50% for sixteen patients who had previous exposure to BCMA–directed bispecific antibodies or CAR-T therapy [27]. Cevostamab (FcRH5×CD3 BsAb), was administered in twenty-eight patients who had received ≥1 prior CAR-T at target dose levels >90 mg, an ORR of 44.4% (4/9 patients) was achieved [28]. MCARH109 (anti-GPRC5D CAR-T) had an ORR of 70% (7/10) for patients who had received previous BCMA therapies [29]. Another anti GPRC5D CAR-T, OriCAR-017 also showed promising results, two patients had a sCR, and three patients had a VGPR in five patients who previously received anti-BCMA CAR-T therapy, including four patients who received murine-derived BCMA-targeted CAR-T and one patient who received sequential murine and human-derived BCMA-targeted CAR-T [30]. Given their efficacy, a reinfusion of alternative anti-BCMA CAR-T is not inferior to these treatments.

In summary, HRC0202 infusion is safe and effective for the R/R MM patients relapsed or refractory after prior CAR-T infusion, nonetheless, further study and more data of a wide variety of other anti-myeloma therapies are needed for the optimal strategies in patients who have relapsed or refractory following anti-BCMA CAR-T therapy.

## Data Availability

Additional data beyond the data found in the article are available from the corresponding author on reasonable request.

## References

[1] Rajkumar SV (2022). Multiple myeloma: 2022 update on diagnosis, risk stratification, and management. Am J Hematol.

[2] Ito S (2020). Proteasome inhibitors for the treatment of multiple myeloma. Cancers.

[3] Derudas D, Capraro F, Martinelli G, Cerchione C (2020). Old and new generation immunomodulatory drugs in multiple myeloma. Panminerva Med.

[4] Bonello F, Mina R, Boccadoro M, Gay F (2019). Therapeutic monoclonal antibodies and antibody products: current practices and development in multiple myeloma. Cancers.

[5] Tandon N, Ramakrishnan V, Kumar SK (2016). Clinical use and applications of histone deacetylase inhibitors in multiple myeloma. Clin Pharm.

[6] Peterson TJ, Orozco J, Buege M (2020). Selinexor: a first-in-class nuclear export inhibitor for management of multiply relapsed multiple myeloma. Ann Pharmacother.

[7] Ali SA, Shi V, Maric I, Wang M, Stroncek DF, Rose JJ (2016). T cells expressing an anti-B-cell maturation antigen chimeric antigen receptor cause remissions of multiple myeloma. Blood.

[8] Munshi NC, Anderson LD, Shah N, Madduri D, Berdeja J, Lonial S (2021). Idecabtagene vicleucel in relapsed and refractory multiple myeloma. N. Engl J Med.

[9] Berdeja JG, Madduri D, Usmani SZ, Jakubowiak A, Agha M, Cohen AD (2021). Ciltacabtagene autoleucel, a B-cell maturation antigen-directed chimeric antigen receptor T-cell therapy in patients with relapsed or refractory multiple myeloma (CARTITUDE-1): a phase 1b/2 open-label study. Lancet.

[10] Wang D, Song Y, Huang H, Li J, Chen B, Liu J (2023). CT103A, a novel fully human BCMA-targeting CAR-T cells, in patients with relapsed/refractory multiple myeloma: updated results of phase 1b/2 study (FUMANBA-1). J Clin Oncol.

[11] Brudno JN, Maric I, Hartman SD, Rose JJ, Wang M, Lam N (2018). T cells genetically modified to express an anti-B-cell maturation antigen chimeric antigen receptor cause remissions of poor-prognosis relapsed multiple myeloma. J Clin Oncol.

[12] Raje N, Berdeja J, Lin Y, Siegel D, Jagannath S, Madduri D (2019). Anti-BCMA CAR T-cell therapy bb2121 in relapsed or refractory multiple myeloma. N Engl J Med.

[13] Rodriguez-Otero P, San-Miguel JF, Anderson LD, Lonial S, Truppel-Hartmann A, Sanford J (2021). Subsequent anti-myeloma therapy after Idecabtagene Vicleucel (ide-cel, bb2121) treatment in patients with relapsed/refractory multiple myeloma from the KarMMa Study. Blood.

[14] Wang D, Wang J, Hu G, Wang W, Xiao Y, Cai H (2021). A phase 1 study of a novel fully human BCMA-targeting CAR (CT103A) in patients with relapsed/refractory multiple myeloma. Blood.

[15] Cheng Z, Wei R, Ma Q, Shi L, He F, Shi Z (2018). In vivo expansion and antitumor activity of coinfused CD28- and 4-1BB-engineered CAR-T cells in patients with B cell leukemia. Mol Ther.

[16] Zhao G, Wei R, Feng L, Wu Y, He F, Xiao M (2022). Lenalidomide enhances the efficacy of anti-BCMA CAR-T treatment in relapsed/refractory multiple myeloma: a case report and revies of the literature. Cancer Immunol Immunother.

[17] Chinese Medical Doctor Association HB, Chinese Society of Hematology CMA. (2022). [The Chinese consensus for the CAR-T cell therapy in multiple myeloma (2022 version)]. Zhonghua Xue Ye Xue Za Zhi.

[18] Gazeau N, Beauvais D, Yakoub-Agha I, Mitra S, Campbell TB, Facon T (2021). Effective anti-BCMA retreatment in multiple myeloma. Blood Adv.

[19] Cui R, Li P, Li Q, Mu J, Jiang YL, Jiang YY (2021). Humanized BCMA CAR-T cell salvage therapy in two refractory multiple myeloma patients who progressed after their murine BCMA CAR-T cell therapy. Zhonghua Xue Ye Xue Za Zhi.

[20] Mailankody S, Matous JV, Chhabra S, Liedtke M, Sidana S, Oluwole OO (2023). Allogeneic BCMA-targeting CAR T cells in relapsed/refractory multiple myeloma: phase 1 UNIVERSAL trial interim results. Nat Med.

[21] Ri M, Suzuki K, Ishida T, Kuroda J, Tsukamoto T, Teshima T (2022). Ciltacabtagene autoleucel in patients with relapsed/refractory multiple myeloma: CARTITUDE-1 (phase 2) Japanese cohort. Cancer Sci.

[22] Holland EM, Molina JC, Dede K, Moyer D, Zhou T, Yuan CM (2022). Efficacy of second CAR-T (CART2) infusion limited by poor CART expansion and antigen modulation. J Immunother Cancer.

[23] Lamers CH, Willemsen R, van Elzakker P, van Steenbergen-Langeveld S, Broertjes M, Oosterwijk-Wakka J (2011). Immune responses to transgene and retroviral vector in patients treated with ex vivo-engineered T cells. Blood.

[24] Samur MK, Fulciniti M, Aktas Samur A, Bazarbachi AH, Tai YT, Prabhala R (2021). Biallelic loss of BCMA as a resistance mechanism to CAR T cell therapy in a patient with multiple myeloma. Nat Commun.

[25] Sebag M, Raje NS, Bahlis NJ, Costello C, Dholaria B, Solh M (2021). Elranatamab (PF-06863135), a B-cell maturation antigen (BCMA) targeted CD3-engaging bispecific molecule, for patients with relapsed or refractory multiple myeloma: results from magnetismm-1. Blood.

[26] Touzeau C, Krishnan AY, Moreau P, Perrot A, Usmani SZ, Manier S (2022). Efficacy and safety of teclistamab (tec), a B-cell maturation antigen (BCMA) x CD3 bispecific antibody, in patients (pts) with relapsed/refractory multiple myeloma (RRMM) after exposure to other BCMA-targeted agents. J Clin Oncol.

[27] Chari A, Minnema MC, Berdeja JG, Oriol A, van de Donk N, Rodriguez-Otero P (2022). Talquetamab, a T-cell-redirecting GPRC5D bispecific antibody for multiple myeloma. N. Engl J Med.

[28] Trudel S, Cohen AD, Krishnan AY, Fonseca R, Spencer A, Berdeja JG (2021). Cevostamab monotherapy continues to show clinically meaningful activity and manageable safety in patients with heavily pre-treated relapsed/refractory multiple myeloma (RRMM): Updated Results from an Ongoing Phase I Study. Blood.

[29] Mailankody S, Devlin SM, Landa J, Nath K, Diamonte C, Carstens EJ (2022). GPRC5D-Targeted CAR T cells for myeloma. N Engl J Med.

[30] Zhang M, Wei G, Zhou L, Zhou J, Chen S, Zhang W (2023). GPRC5D CAR T cells (OriCAR-017) in patients with relapsed or refractory multiple myeloma (POLARIS): a first-in-human, single-centre, single-arm, phase 1 trial. Lancet Haematol.

